# Evaluating the validity of testimony: The role of the order of evidence

**DOI:** 10.1016/j.fsisyn.2024.100562

**Published:** 2024-11-21

**Authors:** Henry Otgaar, Tamara L.F. De Beuf, Melanie Sauerland, Alexa Schincariol

**Affiliations:** a Faculty of Law and Criminology, Catholic University of Leuven, Belgium; b Faculty of Psychology and Neuroscience, Maastricht University, the Netherlands; c Netherlands Institute for the Study of Crime and Law Enforcement, the Netherlands; d Padova Neuroscience Center (PNC), University of Padova, Padova, Italy; e Department of General Psychology, University of Padova, Padova, Italy

**Keywords:** Cognitive bias, Expert witness, Legal psychology, Linear sequential unmasking, Statement validity

## Abstract

Legal practitioners sometimes ask psychologists to evaluate the validity of statements of victims, witnesses, and suspects. For their assessment, psychologists often have access to different pieces of evidence (e.g., a video recording of the interview, the suspect's statements). Research has demonstrated that the order of reviewing the evidence can affect decision-making. To examine expert witnesses' views about this, we surveyed 52 legal psychologists about their preferred order for considering the evidence in a statement validity assessment in a fictional sexual abuse case. The assessment was about the validity of the statement of the alleged child victim. The case file included the following documents: an audiovisual recording of the child interview at the police station, a verbatim transcript of that same interview, and a written statement of the suspect. Legal psychologists indicated their preferred order for reviewing these documents and explained the rationale behind their choice. There was no uniform approach among legal psychologists. About one third of respondents would first examine the audiovisual recording, then the verbatim transcript and finally the suspect's statement. In contrast, about one third would first look at the verbatim transcript, then at the recording and last at the suspect's statement. These differences in approach likely highlight the challenges and trade-offs entailed in deciding on the optimal order and emphasize the need for a discussion in the expert witness community about these issues.

## Highlights

• Fifty-two legal psychologists were asked about the order in which they would examine the evidence in a statement validity assessment.
• There was no uniform approach among legal psychologists.
• Our study points to the lack of best practices and standards on the optimal order for examining evidence.

Psychologists sometimes evaluate the validity of statements of witnesses, victims, or suspects. A crucial task of expert witnesses is to determine whether statements are based on an authentic experience or not [1,2]. Specifically, expert witnesses frequently search for elements in the case file that support or jeopardize the validity of statements. If, for example, an expert witness finds evidence that the police interrogated a suspect with suggestive methods and that a confession was made only after a suggestive interrogation, there are reasons to assume that the confession was coerced [3]. This casts doubt on the validity of the confession. Similarly, an expert witness may discover that an alleged victim disclosed abuse following several therapy sessions involving methods that could lead to memory distortion [4]. Such methods could have affected the validity of the testimony.

When expert witnesses are involved in statement validity assessment, they usually receive a case file with a variety of different types of evidence, such as a statement from a victim, transcript or video recording of the interview with the police, or a record of the suspect interrogation. In the current study, we examined in which order expert witnesses inspect the evidence before reaching a conclusion, and which arguments they provide in support of their preferred order in statement validity assessment.

## 1 The relevance of order

There is a long research tradition showing that the order of information presentation can affect cognition. For example, a well-documented finding is the *primacy effect* [5,6], a phenomenon whereby people remember the information presented first better than the information presented in the middle. The same applies to information that is provided at the end, which is known as the *recency effect* [7]. Not only do these results have theoretical significance, but they also have practical relevance, for example, when child protective agencies or the police interview alleged victims of abuse who have endured repeated traumatic experiences (e.g. Ref. [8]). Evidence-based recommendations dictate that interviewers should ask child victims about the most recent abusive experience, because such experiences are remembered best [9,10].

Apart from the fact that we remember recent information better, information that is perceived first can also have powerful effects on the processing of subsequent information. It can affect the perception and evaluation of subsequent information by inducing selective attention, it can create tunnel vision, and many other well-established cognitive effects (e.g., Ref. [11,12]). For example, in one study, students who had to take the role of jurors and delivered a final verdict were affected by a tentative verdict they had provided earlier [13]. Specifically, students who gave an innocent tentative verdict tended to vote acquittal at the final verdict, while students who initially gave a guilty verdict were more likely to convict a suspect.

In another study, students received different types of information (confirming, disconfirming) from different sources (experts, friends) in various order [14]. The information concerned a possible upcoming military attack. Following this information, students had to estimate the probability of an attack. Confirming information from an expert in an initial position enhanced the estimated probability of an attack while disconfirming information from a friend in an initial attack decreased it. These effects, in which the order of information provision affects decision-making, are referred to as *order effects*. As with other biases, they are part of human cognitive processes and, therefore, apply to all experts who engage in some sort of decision-making, including forensic experts, medical experts, police investigators, and also legal psychologists [15].

## 2 Cognitive bias

Cognitive bias refers to a systematic (unconscious) deviation in “normal” decision-making and is present in everyone [16]. While cognitive biases can facilitate the processing of information, they can also have adverse consequences. For example, when people are interviewed by the police about an event that happened months ago (e.g., witnessing a bank robbery), their memory might bias them to remember details consistent with the (general idea of the) event that were actually not present (e.g., memory of seeing a gun while in fact it was a knife). This can occur as a result of cognitive phenomena, such as memory reconstruction (Loftus, 2005).

Bias among (forensic) experts can affect legal outcomes, cumulating in the wrongful conviction of innocent suspects (e.g. Ref. [17]). For example, handwriting experts can be biased in their assessment by external information irrelevant to their task. That is, handwriting experts who are informed about a suspect's confession are more likely to conclude that the signature found at the crime scene was from this suspect, while it may well be the case that the confession was false [18]. This biased decision-making is referred to as *forensic confirmation bias* and has been observed in various forensic disciplines, such as forensic pathology, forensic DNA analysis, and fingerprint analysis ([3]; for a review, see Ref. [12,19]).

Psychologists who evaluate the validity of statements are also not immune to bias. For example, when psychologists are hired by the defense or the prosecution, the side that retains them can bias how they evaluate the evidence, an effect termed *allegiance bias* ([20]; see also [21]). Specifically, students who were instructed to act as an expert witness and assess the validity of a victim statement of alleged abuse, were more likely to find the testimony valid when they were hired by the prosecution than by the defense [21].

Such biases do not only affect the evidence that is examined. Rather, their effects can spill over to the entire case, what is known as the *bias cascade* and *bias snowball* effects [22]. The consequences of cognitive bias mandate that expert witnesses apply methods to minimize this bias in decision-making [1,2,23].

## 3 Bias minimization

To minimize bias due to the order of evidence, experts can consciously regulate the order. Specifically, *Linear Sequential Unmasking-Expanded* (LSU-E) is a method proposed to reduce bias by controlling the flow of information to forensic experts ([24]; but see also [25], in press). The underlying idea of the LSU-E is rather straightforward. Forensic experts should only be exposed to information that is relevant to their task, and should always start with the actual data/evidence before receiving any other task-relevant information. For example, a handwriting expert should start with the actual document for which the evaluation was requested before being exposed to the suspect's handwriting and performing the comparison. The aim of regulating this order is obvious. Forensic experts should first form an opinion based on the actual data or evidence (e.g., letter forms) before considering any other relevant (contextual) information.

LSU-E also includes specific criteria on how to optimize the order of information, namely biasing power, objectivity, and relevance [24,26]. When considering the most optimal flow of information, forensic experts should first start with information that is the least biasing, most objective, and most relevant for the case at hand. For example, a forensic pathologist should first examine the body of the deceased, because this piece of evidence is objective, relevant and with minimal bias. Only after this examination, the expert should inspect other relevant information such as the personal background of the victim (e.g., medical history).

Importantly, the goal of LSU-E is not to prevent forensic experts from receiving any contextual relevant information. In some forensic disciplines, there will usually be some exposure to relevant contextual information. Take for example a legal psychologist who evaluates the validity of testimony. When undertaking this task, a legal psychologist will consider different pieces of evidence, such as statements from witnesses, suspects, and alleged victims. Exposure to context information is inevitable, for instance, exposure to the victim and suspect's race when viewing the video recording (see Ref. [22]). In other situations, it may be revealed that a witness knows the main suspect and discloses personal information about the suspect during an interview (e.g., previous criminal acts; see Ref. [22]).

In many cases regarding the validity of testimony, objective evidence is absent (e.g., photos of abuse), meaning that the ground truth is unknown. Legal psychologists can use other available evidence to evaluate the validity of testimony. It is not clear, however, whether legal psychologists sufficiently consider the importance of order when evaluating the available evidence to reach a conclusion about the validity of statements. When assessing different pieces of evidence, some evidence might be more biasing, less relevant, or less objective than other types of evidence. For example, Dror and Kukucka [24] argued that “an eyewitness account of an event is typically less objective than a video recording of the same event” (p. 3) and that “a statement from a sober eyewitness may be considered before a statement from an intoxicated witness” (p. 4).

Legal psychologists frequently receive various types of evidence such as (video) recordings of a police interview with alleged victims, suspects, and witnesses, and transcripts of interviews with alleged victims, suspects, and witnesses [2]. Drawing on the tenets of LSU-E, when legal psychologists evaluate the validity of an alleged victim's statement, one might argue that they should first look at the recording(s) of interviews with the victim prior to evaluating the transcript of these interviews and interviews with others (e.g., suspect). The rationale underlying this is that the recording of the interview with an alleged victim can be regarded as the most objective, most relevant, and likely least biasing piece of evidence. Given that the interviews are the evidence and the transcript is in some occasions an interpretation of the interview (i.e., no verbatim transcript), it would be advised, from the cognitive bias literature, that the interview recording is seen prior to the transcripts (for details, see Ref. [24]).

## 4 The current study

In the current study, we surveyed legal psychologists who are involved in statement validity assessment in court cases. Specifically, we presented them with a fictitious case concerning child sexual abuse containing three pieces of evidence: a video recording of the interview with the alleged victim, a transcript of the interview with the alleged victim, and a statement from the suspect. We asked them which order they would use when evaluating the evidence. Since there are no standards or guidelines on this matter, we were interested in whether there was a consistent order that psychologists would use when working on this case.

## 5 Method

### 5.1 Respondents

We invited legal psychologists to participate through various channels. First, we created a list from our own network of legal psychologists who are involved in statement validity assessment (*n* = 41). Second, we emailed them to participate in our study and asked whether they had any suggestions for other possible contacts, which led to an additional 11 respondents. Third, we obtained email addresses from German legal psychology websites in which legal psychologists are listed and contacted them to participate in our study.^1^ Fourth, the *European Association of Psychology and Law* and the *Society for Applied Research in Memory and Cognition* emailed their members an invitation to participate in our study. These recruitment strategies led to a sample size of 52 legal psychologists (mean age^2^ = 51.57, *SD* = 14.70; range 25–82; 24 male). The current study was approved by the standing ethical committee of the Faculty of Psychology and Neuroscience, Maastricht University (ERCPN-264_23_02_2023). All data and materials can be accessed via this link: https://osf.io/kzf5j/.

### 5.2 Materials

Respondents received a survey with instructions followed by several questions. First, they imagined that they had been recruited to provide written expert testimony in a case of child sexual abuse. Second, it was stated that they had to evaluate the validity of the statement of the alleged victim, a young child. Third, they learnt that the case file contained several documents.

1. A verbatim transcript of the child interview at the police;
2. An audiovisual recording of the child interview at the police;
3. A written statement of the suspect.

Next, respondents determined in which order they would consider the available evidence for their assessment of the validity of the child's testimony. There were eight response options respondents could choose from: “no specific order”, “depends on the case”, and six options representing all possible order variations. Finally, respondents answered two open-ended questions inviting them to explain the rationale for their choice and what they considered the most relevant information in a case file when evaluating the validity of statements.

### 5.3 Procedure

The survey was created using Qualtrics software (http://www.qualtrics.com). Respondents received a link to the online survey. The survey started with a letter containing general information about the study (e.g., short description about the procedure, information about privacy). Then they received an informed consent form. If they agreed to participate, they viewed the description of the fictitious case on alleged child sexual abuse (see Materials). After answering several questions about the case, respondents indicated their gender, age, how many cases they had worked on as an expert witness, how many years they had served as an expert witness for, current employment, level of education, and country of residence. Finally, respondents received a debriefing.

## 6 Results

### 6.1 Background information

Respondents had worked on a mean of 118.73 (*SD* = 157.04) statement validity cases as an expert witness and had a median of 14 years of experience as an expert witness. Most respondents worked at a university but about a third of respondents were self-employed (see https://osf.io/kzf5j/?view_only= for more details). Our respondents also came from diverse set of regions in the world (see https://osf.io/5dpky).

### 6.2 The order of evidence

There was no uniformity in the preferred order. Instead, about a third of respondents indicated that they would first look at the verbatim transcript of the child interview, then at the audiovisual recording of that interview, and finally at the written statement of the suspect. An almost similar percentage of subjects chose first to look at the audiovisual recording of the child interview, then the verbatim transcript of the interview, and finally at the suspect's statement (see also Table 1).

**Table 1:** Respondents’ preference of the order of evidence.

Order	Number (percentage)
I would first look at 1, then 2, and finally 3	19 (36.5)
I would first look at 2, then 1, and finally 3	16 (30.6)
I would first look at 1, then 3, and finally 2	5 (9.6)
The order depends on the type of case	4 (7.7)
I do not use a specific order	3 (5.8)
I would first look at 2, then 3, and finally 1	2 (3.8)
I would first look at 3, then 1, and finally 2,	2 (3.8)
I would first look at 3, then 2, and finally 1	1 (1.9)

*Note*. 1 = verbatim transcript of child interview, 2 = audio/visual recording of child interview, 3 = suspect's statement.

### 6.3 Rationale and most relevant information

Using a more qualitative approach, we assessed the respondents' answers to the open-ended questions. We did not use a structured approach as this analysis was purely exploratory. First, we investigated the reasons why they decided to look at a particular evidence first. Respondents who would first consider the transcript of the child interview chose this piece of evidence, because it would provide them with an overview and a first impression of the child's statement. Furthermore, they indicated that they first wanted to read the transcript because this would enable them to assess the quality of the interview and compare it with the audiovisual recording.

Respondents who chose to start their evaluation by looking at the video recording of the child interview stated that this piece of evidence was original and the most clear and accurate evidence. They also noted that such a recording would provide an unbiased depiction of the interview. Interestingly, respondents who did not select a recording as the first piece of evidence mentioned as a reason that the recording might actually bias the expert witness. For example, they stated that the emotions displayed by the alleged victim could affect the expert's opinion. Lastly, respondents who wanted to first evaluate the suspect's statement posited that this statement would be helpful in generating alternative hypotheses concerning the child's allegation.

Furthermore, we examined what respondents, in general, considered to be relevant information when evaluating the validity of statements. Respondents provided various answers, such as the quality of the interview, circumstances of the disclosure, and other factors affecting the statement (see Table 2).

**Table 2:** Relevant information for assessing the validity of statements according to respondents.

Relevant information	Number of respondents
Quality of the interview	24
Information about the child	23
Circumstances of the first disclosure	21
Consistency of statements	18
Factors influencing the statement	14
Information from other witnesses	11
Other forensic evidence	11
Other evidence related to the alleged victim	10
Suspects' statements	10
Timeline	8
Statement validity assessment (e.g., CBCA)	6
Recovered memory	4
Possible grounds for false testimony	4
Plausibility of the event	4

*Note*. Respondents could provide several pieces of information of relevance for statement validity assessment, so the numbers do not add up to the sample size of the respondents.

## 7 Discussion

Psychologists sometimes provide expert testimony on the validity of statements of victims, witnesses, and suspects (e.g. Ref. [23]). Basically, expert witnesses are consulted to examine whether a statement refers to an authentic experience. To undertake such an investigation, expert witnesses access different pieces of evidence such as forensic victim interviews by the police, suspect statements, and eyewitness testimony. The purpose of the present study was to examine in which order legal psychologists would review these pieces of evidence.

This issue of order is important because the sequence in which information is considered, can affect decision-making (e.g., Ref. [24,27]). We therefore asked legal psychologists working on statement validity assessment, which order they would choose when evaluating the validity of a child's testimony. The main finding was that legal psychologists did not agree on the preferred order of reviewing the evidence. The opinions mostly differed with respect to whether they would review the transcript or the audiovisual recording first. Some argued that the audiovisual recording should be prioritized, because it was the most accurate and unbiased piece of evidence and because it was not tainted by the interpretation of the interviewers or transcribers. Others argued that the audiovisual recording should succeed the transcript, because the audiovisual material contained potentially biasing information that was not present in a transcript, such as emotions of the alleged victim.

These differences in approach highlight the challenges and trade-offs entailed with deciding on the optimal order. In the scientific literature, strategies have been suggested to assist in determining the evidence sequence, such as linear-sequential unmasking (LSU-E). According to the framework, the order for reviewing evidence should be cognitively informed. That is, the least biased, most objective, and most relevant piece of evidence should be examined first [24]. Applying these criteria to the situation presented in this study, we argued that legal psychologists should first consider the audiovisual recording of the victim's interview, then proceed with the verbatim transcript of the child's interview, and end with the suspect's statement. However, less than one third of the respondents followed this reasoning.

If we assume that respondents were aware of the importance of order, they must have decided on an alternative order because they assigned different biasing power to the evidence. For example, they might have reasoned that an audiovisual recording could be especially biasing. This issue is in line with research showing that confessions are evaluated as more voluntary when the camera footage shows primarily the suspect than when the suspect is seen from different camera angles (e.g., when the camera footage shows the interrogator and suspect [28]). However, in other situations, audiovisual recordings are not particularly biasing. For example, research on the emotional child witness effect demonstrates that children are perceived as most credible when they appear sad and that this effect is equally strong when respondents rate the credibility of children in interview transcripts, audio, or visual recordings [[29], [30]]. Other reasons for not starting with reviewing the audiovisual recordings include that it might be too costly and time consuming to look at them in cases where suspects are interviewed for hours and repeatedly, resulting in a vast amount of recorded testimony. In these cases, it can be nearly impossible to critically evaluate the recordings, given restrictions in time and financial resources. Hence, in these situations, expert witnesses might decide to start with the verbatim transcripts when evaluating the validity of statements.

Respondents' responses to the open-ended question further inform about their motivation for reviewing the transcripts first. They reasoned that transcripts contained less biasing information than audiovisual material. It is likely that experts who rely on this argument assume that the transcript is an exact reproduction of what has been said in the interview. However, such ‘verbatim’ transcripts are not always in line with the actual recordings [[31], [32], [33]].

More surprising is the decision of few experts (about 6 %) to start with the suspect's statement. As a rationale, respondents mentioned that assessing the suspect's statement first would be helpful in creating alternative hypotheses on the validity of the victim's statement. Generating alternative hypotheses is indeed commendable as a strategy for reducing cognitive biases in legal psychological assessments [1,2]. More specifically, this recommendation – also referred to as the *(alternative) scenario method* – advises legal psychologists to work with multiple scenarios. It suggests that experts should look for evidence that supports the scenario in which a statement is valid *as well as* evidence that is in line with the alternative scenario, namely that the statement is invalid [34,35]. Respondents might have prioritized this method above and beyond other potential debiasing methods such as the importance of the order of evidence. It is an empirical question to investigate whether the *scenario method* or LSU-E is superior to minimize cognitive bias in statement validity assessment.

Taken together, legal psychologists did not make a uniform decision with respect to the order in which they would assess the evidence in a case. This indicates that awareness of and discussion on the topic is needed in the field of legal psychology. Such debate may start with an inventory of which information is (most) relevant versus irrelevant to the task at hand. Albeit fundamental, this question may generate discussion and input for future research, given the diversity of responses we received on the open-ended question. In line with this, research with forensic technical experts has demonstrated that there is considerable variability within disciplines on what experts consider essential to their analysis (see Ref. [36]). Moreover, it may be even more difficult to establish what constitutes “relevant” case information in legal and forensic psychology than in technical forensic disciplines [37]. Nevertheless, given the potential impact of evidence order and the bias-mitigating potential of strategies that address evidence order, it is a conversation the field should open. The current study is a first contribution to such conversation.

### 7.1 Limitations

Our study is limited in the sense that the surveyed legal psychologists were not involved in a real court case and they were only asked about their preferred order regarding a limited list of evidence. If legal psychologists were involved in an actual case, they likely would have received other and more pieces of evidence. For example, in real cases, they might have encountered interviews with others, such as testimony from parents or background information concerning the suspect and alleged victim. These other pieces of evidence might all differ on levels of relevance, biasing power, and objectivity and hence, could affect the order that psychologists use when evaluating the validity of statements.

### 7.2 Recommendations for practice

Echoing previous recommendations regarding expert work [2], we advise that legal psychologists could report transparently about the evidence they used during their assessment, the order in which they considered the evidence, and why. This in and of itself may not minimize bias but will increase transparency about the decision-making process. Moreover, in an ideal scenario, each time a new piece of evidence is examined, legal psychologists should document whether their evaluation and preliminary conclusion changed in any way. This approach to expert witness reporting improves transparency and allows others to validate the expert's decision-making process [24,26]. We do not mean to imply that legal psychologists who have not described the sequence of reviewing the evidence have violated any expert witness guidelines. However, we do emphasize the need for a discussion in the legal psychological community about this issue.

## 8 Conclusion

The current study sheds light on the diversity of practices among legal psychologists when evaluating the validity of statements from an alleged sexual abuse child victim. We investigated how legal psychologists handled evidence and, more specifically in which order they examined the information in a fictitious case. The research revealed variability in the order in which experts decided to examine the available documents, including verbatim transcripts of child interviews, audiovisual recordings, and a written suspect statement. The absence of a consensus on the optimal sequence for reviewing evidence highlights the need to increase awareness on the optimal order of evidence in legal psychological assessments.

## CRediT authorship contribution statement

**Henry Otgaar:** Writing – review & editing, Writing – original draft, Project administration, Methodology, Investigation, Formal analysis, Conceptualization. **Tamara L.F. De Beuf:** Writing – review & editing, Methodology, Conceptualization. **Melanie Sauerland:** Writing – review & editing, Methodology, Conceptualization. **Alexa Schincariol:** Writing – review & editing, Methodology, Formal analysis, Conceptualization.

## Funding

Funding was received by funding agency: 10.13039/501100003130Fonds Wetenschappelijk Onderzoek (FWO). Grant number: G0D3621N.

## Declaration of competing interest

The authors declare that they have no known competing financial interests or personal relationships that could have appeared to influence the work reported in this paper.

## Footnotes

1 See https://www.gwg-institut.com/sachverstaendige/aussagepsychologische, https://zwpd.transmit.de/zwpd-dienstleistungen/zwpd-rechtspsychologie/zwpd-supervisoren-fachteams-pruefer.

2 There was one participant who indicated to be six years old. This participant was removed from the analysis on age but was included for the rest of the analyses as the participant provided plausible answers to the open question and hence likely made a typo when inserting their age.
